# Assessment of Tetracyclines Residues and Tetracycline Resistant Bacteria in Conventional and Organic Baby Foods

**DOI:** 10.3390/foods4030306

**Published:** 2015-07-22

**Authors:** Mónica Guarddon, José M. Miranda, Beatriz I. Vázquez, Alberto Cepeda, Carlos M. Franco

**Affiliations:** Department of Analytical Chemistry, Nutrition and Bromatology, Faculty of Veterinary, University of Santiago de Compostela, Carballo Calero st., s/n. 27002-Lugo, Spain; E-Mails: monica.guarddon@gmail.com (M.J.); josemanuel.miranda@usc.es (J.M.M.); beatriz.vazquez@usc.es (B.I.V.); alberto.cepeda@usc.es (A.C.)

**Keywords:** tetracycline-resistance, baby foods, qPCR, *tet*(A) gene, *tet*(B) gene

## Abstract

Children are very vulnerable to bacterial infections and they are sometimes subject to antimicrobials for healing. The presence of resistance genes may counteract effects of antimicrobials. This work has thereby compared the amount of tetracycline resistance genes, *tet*(A) and *tet*(B), between conventional and organic meat-based or vegetable-based baby foods and used the quantification of these genes to assess the presence of tetracycline residues in these samples. Counts of bacteria harboring the *tet*(A) gene were higher than those containing *tet*(B), and there was no difference between the organic and the conventional samples. Samples with detectable amounts of tetracycline residues were also positive for the presence of *tet* genes, and when the presence of the genes was not detected, the samples were also negative for the presence of residues. The percentages of tetracycline residues were higher in organic samples than in conventional ones. It cannot be concluded that organic formulas are safer than conventional ones for the studied parameters.

## 1. Introduction

The use of antimicrobials in infants and children is quite controlled because this population group is very susceptible to bacterial infections; their immune systems are not completely developed [1], and some of these agents are still not adequate for them. One example is the use of tetracycline (Tc) in children, which can permanently damage the enamel of their teeth [2]. Additionally, recent investigations revealed that antimicrobial use in early life is associated with consistent increases in body mass and could therefore contribute to the increase in childhood obesity [3].

Animal infectious diseases are frequently treated with antimicrobial agents, which contribute to the development of resistant bacteria that could pose a human health hazard through the food chain [4,5,6]. As a consequence, conventional farming is not favorably regarded due to the crowding conditions of the animals on farms, which facilitate the appearance of infectious diseases and, therefore, encourage the disproportionate use of antimicrobial agents [7]. Contrary to this type of animal rearing, Regulation 889/2008/EC provides details of the restrictive rules for obtaining organic products, which are guaranteed by the community logo on the label of the product [8]. Thus, in regards to sickness encountered in organic farming, synthetic allopathic medicines should be limited to the minimum number possible, and the withdrawal period must be twice the established time for conventional production. Because of this regulation, organic products have become an attractive option for consumers who often perceive these products as being healthier and safer than the products obtained from conventional farming [9,10].

One of the antimicrobials most used in Europe for the treatment of animal infections is the Tetracyclines (Tc) group [11]. Notably, in Spain, Tc was the best-selling antimicrobial family in 2012 [12]. The indiscriminate use of these agents has favored the selection and distribution of Tc-resistant bacteria [13]. The majority of Tcr (*tet*) genes in bacteria have been associated with genetic elements, which facilitate the rapid dissemination of these genes among bacterial species [13,14]. Likewise, a great amount of free genes in the environment can contribute to bacterial transformation, increasing the spread of tetracycline-resistant bacteria. A review of DNA uptake mechanisms by bacteria can be found in the scientific literature [15]. Nevertheless, due to the reduced antimicrobial selection pressure in organic farming, it is reasonable to think that Tc-resistant bacterial counts should be higher in conventional products than in organic products. In this sense, a lower presence of methicillin resistant *Staphylococcus aureus* in organic farming as compared with conventional farming was reported recently [16]. Because bacterial antimicrobial resistance could reduce the number of effective drugs available to treat sick infants and children, this issue is important (Keep antibiotics working, 2002). It would be interesting to know if organic baby foods are a healthier alternative to conventional baby foods as organic food is expected to have fewer Tc residues and, therefore, fewer Tc-resistant bacteria.

To date, the majority of the reported methods for the analysis of residues of veterinary drugs in baby foods involve long sample preparation and analysis [1,17]; simpler methods are required to control these special foods.

In the present work the *tet*(A) and the *tet*(B) genes have been used for the first time as biomarkers to quantify, by quantitative PCR (qPCR), the Tc-resistant bacteria in organic and conventional meat-based baby foods because these genes are two of the most frequently found *tet* genes in Gram-negative bacteria [13]. Taking the nature of the samples into account, the main objective of the present work is to compare the obtained results between the conventional and the organic samples and among the tested species. Furthermore, a secondary objective was to investigate the correlation between the amount of bacteria harboring the cited genes and the amount of Tc residues in the baby food using a receptor assay.

## 2. Experimental Section

### 2.1. Sample Collection

A total of 151 samples of conventional (83) and organic (68) baby foods for infants and young children were analyzed in the present work. The choice of the samples, in the form of puree, was made according to the composition: 67 with poultry meat (38 conventional and 29 organic), 67 with beef (34 conventional and 33 organic) and 17 vegetable baby foods (11 conventional and 6 organic). All of the samples were bought in supermarkets and pharmacies in Galicia (North-Western Spain), Madrid (Spain), Dresden (Germany) and Milan (Italy).

The composition of the different samples according to the labels was based on poultry meat (chicken or turkey meat) (4.5%–40%), beef (8%–40%) and vegetable-based baby foods, in which case the content varied from 67% to 99%. Other ingredients such as rice, pasta or vegetables, among others, were also integrated in the sample. The presence of milk was only reported in the label of some chicken meat-based products (18%).

The number of trademarks varied according to the animal species used and the farming method used. Thus, the research was carried out with eight trademarks of conventional beef and six of organic beef, seven of conventional chicken meat and eight of organic chicken meat and five trademarks of vegetable samples. Samples purchased in the same establishment were obtained on different days, and all samples belonged to different batches. Afterward, the samples were transported to the laboratory and were analyzed within 48 h of collection.

### 2.2. Sample Preparation for qPCR

Portions of 35 g from each sample were taken and added to 315 mL buffered peptone water (Merck, Darmstadt, Germany) in a sterile bag with a lateral filter. Samples were homogenized in a masticator MIX 2 (AES, Combourg, France) for 2 min. Aliquots of 200 µL of the homogenates were subjected to DNA isolation using the High Pure PCR Template Preparation Kit (Roche, Mannheim, Germany), according to the manufacturer’s instructions for the isolation of nucleic acids from bacteria or yeast. After isolation, purified DNA was recovered in 50 µL of elution buffer and stored at −20 °C until PCR analysis.

### 2.3. PCR Conditions

Primers, probes previously designed by Guarddon *et al.* [18] and Environmental Master Mix 2.0 (containing ROX as a passive reference) were obtained from Applied Biosystems (Warrington, UK). qPCR was performed using 25 µL reaction volumes, which included 7.5 µL template DNA, 12.5 µL Environmental Master Mix, 900 nmol each primer (forward and reverse) and 200 nmol Taqman probe. Amplification, detection and quantification were performed using an ABI PRISM 7000 Sequence Detection System (Applied Biosystems) under the following conditions: 10 min at 95 °C for Taq-polymerase enzyme activation, followed by 40 cycles of 15 s at 95 °C for denaturation and 1 min at 60 °C for annealing and extension. Standard curves and food samples were processed in duplicate. Negative controls, consisting of all of the elements of the reaction except for the template DNA, were included in all tests.

### 2.4. Quantification Assays

The steps to create the standard curves were based on the method reported by Guarddon *et al.* [18]. To quantify bacteria harboring the *tet*(A) gene, an aliquot of each type of baby food was artificially inoculated with *Escherichia coli* BM13 (C600 RifR)/RP4, provided by the Institute Pasteur (Paris, France), whereas *Escherichia coli* NCTC 50,365, obtained from the National Culture Type Collection (NCTC) (Health Protection Agency, Salisbury, United Kingdom), was used for the *tet*(B) gene. Both strains were grown at 41 °C in Violet Red Bile Glucose Agar (VRBG) (Merck) for 24 h. After incubation, Brain Heart Infusion (BHI) (Difco, Detroit, MI, USA) tubes were inoculated with these isolated strains and were subsequently incubated at 31 °C to obtain a content of 10^9^ CFU·mL^-1^, which was determined by a McFarland densitometer (Dinko, Barcelona, Spain) and confirmed by plate counting in Plate Count Agar (Merck).

Standard curves were created using 10-fold serial dilutions from the cited pure cultures, which carry both the tetA or tetB gene, ranging from 10^9^ CFU·mL^−1^ to 10^2^ CFU·mL^−1^. A portion of 35 g from the puree was aseptically added to 315 mL of buffered peptone water (Merck) in a sterile bag with a lateral filter and homogenized in a masticator for 1 min. Then, five tubes were filled with 9 mL of the homogenate, and each tube was filled with 1 mL of each dilution of the pure cultures, from 10^6^ CFU·mL^−1^ to 10^2^ CFU·mL^−1^ (corresponding to a final content from 10^7^ CFU·g^−1^ to 10^3^ CFU·g^−1^).

For quantifying Tc-resistant bacteria in the samples, 35 g aliquots were added to 315 mL of buffered peptone water in a sterile plastic bag with a lateral filter. All of the samples were homogenized in a masticator MIX 2 for 1 min. Additionally; two non-inoculated aliquots were filtered through a syringe filter of 0.45 µm and analyzed as negative controls.

An aliquot of 200 µL from each dilution/sample was subjected to DNA isolation, recovered in 50 µL of elution buffer and stored at −20 °C until PCR analysis. After the qPCR reaction, the cycle threshold (C_T_) was plotted against the log concentration of the template DNA. Samples for the standard curves and food samples were processed in triplicate and duplicate, respectively, and the averaged C_T_ values were calculated in all cases.

### 2.5. Quantification of Tetracyclines Residues in Baby Foods

A receptor assay (Superscreen Tecna, TS, Italy) was used for the determination of Tc residues in samples of baby food. This kit was designed to detect Tc, oxytetracycline, doxycycline and chlortetracycline in meat samples at concentrations according to the Regulation (EC) No 470/2009 [19]. Considering there are no specific enzyme immunoassays for the detection of Tc in baby foods and due to the content of meat in the samples we have carried out the assays according to the manufacturer’s instructions as if the types of baby food were samples of meat. Every sample was analyzed in duplicate, and the results were considered the average of both determinations for each sample. Absorbance was measured with a plate reader (Das, Roma, Italy) at 450 nm.

### 2.6. Statistical Analysis

Comparison between the qPCR counts obtained from conventional and organic baby foods were determined by Student’s *t* test. The differences were considered to be statistically significant when *p* < 0.05. All of the analyses were performed using the PASW (version 18.0) (IBM, Chicago, IL, USA).

## 3. Results and Discussion

All of the standard curves, created from each type of baby food, exhibited a linear relationship between log input CFU·g^−1^ and C_T_, and the values of slope were close to those recommended by Higuchi *et al.* [20]. These values, shown in Table 1, are better than those previously obtained by Guarddon *et al.* [18] because the efficiency of the reaction was improved. Linearity decreased below 3.7 log CFU·g^−1^, which means that minimal amounts of the *tet* genes could be detected, but quantification below this limit may have decreased the accuracy of the results. Thus, the limit of detection established by us for every matrix was 3.7 log CFU·g^−1^ to quantify the amounts with more precision.

**Table 1:** Values of square regression coefficient (R2) and slope of the standard curves.

product	*tet*(A)		*tet*(B)	
	*R*^2^	Slope	*R*^2^	Slope
Poultry meat	0.9997	−3.32	0.9996	−3.29
Beef	0.9887	−3.17	0.9876	−2.97
Vegetables	0.9985	−3.26	0.9747	−3.20

Notes: These results were obtained from the standard curves constructed from baby foods based on poultry meat, beef and vegetables inoculated with *E. coli* BM13 (C600 RifR)/RP4 (*tet*(A)) and with *E. coli* NCTC 50,365 (*tet*(B)).

**Figure 1:**
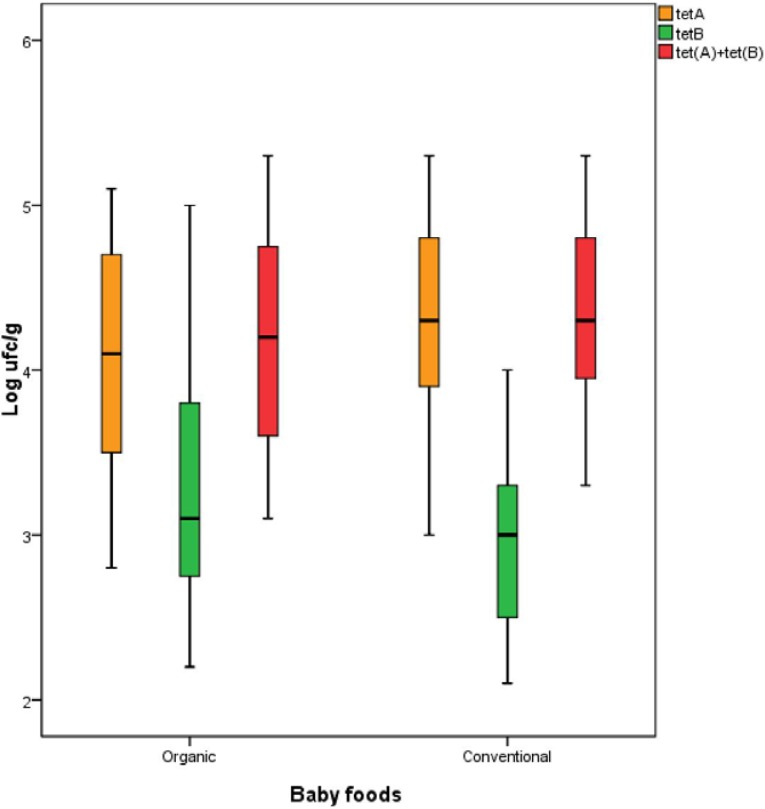
Box plots showing a comparison of the counts of bacteria harboring the *tet*(A) and/or *tet*(B) genes between organic and conventional baby foods. Notes: The box limits are in the 25th and 75th percentile, and the band in the middle of the box is the median; the whiskers are in the 1.5 interquartile range.

Bacteria presumptively harboring the *tet*(A) and/or *tet*(B) genes were quantified with respect to the standard curves. The results showed that, comparing the production methods, counts of *tet*(A) were significantly higher in the conventional products than in the organic products, which would be expected due to the restricted use of antibiotics in organic production (Figure 1). However, in the case of *tet*(B), the counts were significantly greater in the organic samples compared to the conventionally produced samples. No differences were found when the sum of both genes was compared in both types of production. Contrary to other studies in which the prevalence of resistant bacteria were mainly higher in conventional meat [10,21,22], in the present work we have not found clear evidence of this difference for the tetracycline resistance (Tcr) parameter between both types of farming methods. This fact was also highlighted by other researchers such as Wilhelm *et al.* [23], who did not find significant differences between organic and conventional dairy production in four of seven articles, which were cited in their review article about the prevalence of multidrug resistant bacteria.

Regarding the samples containing poultry meat, there were no significant differences between organic and conventional production in the case of the *tet*(A) gene (Table 2). Interestingly, the counts of bacteria presumed to contain *tet*(B) were significantly higher in organic farming. In contrast, with respect to the beef-based samples, the counts of bacteria presumed to harbor the *tet*(A) gene were higher in conventional baby foods than in organic baby foods. However, no significant differences were found when the two types of production systems were compared for *tet*(B). Taking into account the importance of both *tet*(A) and *tet*(B) genes in Gram-negative bacteria, we have also added the results for both genes to make an estimation of their prevalence in this group as well as in other resistant bacterial populations that could be present in the raw material of the baby foods before the heat treatment. Nevertheless, the results showed that there were no significant differences between poultry meat-based conventional and organic samples, whereas samples with beef contained significantly higher Tcr genes in the conventional samples than in the organic samples. These results differ from other studies in which *Enterobacteriaceae* isolated from organic poultry meat [22] or isolates of *E. coli* and *S. aureus* from organic beef [24]. Miranda *et al.* [24] have shown that organic production may limit the presence of antibiotic resistant bacteria in meat. However, in this work, the lack of significant differences between organic and conventional production correlates with the work of Sato *et al.* [25] about the antimicrobial susceptibility of *S. aureus* in organic and conventional milk. These researchers found little differences in antimicrobial resistance patterns between *S aureus* from organic and conventional farms.

**Table 2:** Mean counts of bacteria harboring the *tet*(A) and/or the *tet*(B) genes in conventional and organic baby foods based on poultry meat, beef and vegetables.

Farming Method	Type of Sample	Genes [CFU·g^−1^]			Tetracycline Residues [µg·kg^−1^]
		*tet*(A)	*tet*(B)	*tet*(A) + *tet*(B)	
Conventional	Poultry meat	4.5 ± 0.574 *	3.2 ± 0.526	4.6 ± 0.540	51.2 ± 5.44
	Beef	4.2 ± 0.426	2.7 ± 0.404	4.2 ± 0.400	51.2 ± 6.74
Organic	Poultry meat	4.6 ± 0.471	3.9 ± 0.378	4.7 ± 0.394	53.1 ± 6.39
	Beef	3.7 ± 0.481	2.9 ± 0.415	3.8 ± 0.449	66.2 ± 426
	Vegetables	3.9 ± 0.499	2.5 ± 0.339	3.9 ± 0.485	53.3 ± 13.9

Notes: Mean counts of Tc residues are expressed in micrograms per kilogram of Tc equivalents.* Standard Deviation.

Based on the nature of the samples and regardless the farming method, the amount of Tc-resistant bacteria detected within the poultry meat-based products was significantly higher than the amount detected in beef- or vegetable-based products (Table 3). However, in the beef-based samples, this difference was only observed for the *tet*(B) gene; bacteria harboring this gene were greater in the beef-based food than in the vegetable-based baby foods. Conversely, no differences were found either for the *tet*(A) gene or for the addition of both genes. These results would be expected because Tc are frequently used in animal medicine, which could increase the amount of Tc-resistant bacteria in the animal [6,13]. In fact, the presented data are in accordance with those reported in the Danish Integrated Antimicrobial Resistance Monitoring and Research Programme in 2012 [26], where percentages of Tc-resistant *E. coli* were higher in poultry meat than in beef.

**Table 3:** Mean counts of bacteria harboring the *tet*(A) and/or the *tet*(B) genes in baby foods for infants and young children based of poultry meat, beef and vegetables.

	Vegetables	Beef	Poultry
Tet A	3.9 ± 0.50	3.9 ± 0.53	4.6 ± 0.53
Tet B	2.5 ± 0.34	2.8 ± 0.42	3.5 ± 0.57
Tet (A) + Tet (B)	3.9 ± 0.49	4.0 ± 0.48	4.6 ± 0.49

Mean counts in log UFC·g^−1^ equivalents and Standard Deviation of the data.

In all types of baby foods, the counts of bacteria harboring the *tet*(A) gene were significantly higher than *tet*(B) in both types of production (Table 2). This result is in accordance with other authors who also found the presence of *tet*(A) more frequently than *tet*(B) in porcine *E. coli* [27,28], *E. coli* isolated from foods of animal origin [29], *E. coli* isolated from food animals [30] *and* Gram-negative clinical isolates [31]. Guarddon *et al.* [32] also detected more *tet*(A) genes that *tet*(B) in samples of meat. However, it is necessary to highlight that the data presented in this work were obtained from the total microbiota in the baby food samples rather than from isolates, which offers a complete perspective on the amount of bacteria that harbor these genes. This difference is a highly relevant issue because, although almost all of the bacteria present in these types of samples are dead, extracellular segments of DNA with *tet* genes could be transmitted by horizontal transfer to other living bacteria [33,34], such as the intestinal microbiota of babies. Moreover, it must be noted that the quantification of the cited genes serves as an indicator of the raw material in the samples before being processed.

In the case of vegetable-based baby foods, mean counts of bacteria harboring the *tet*(A) gene were also higher than those harboring the *tet*(B) gene. The sum of both genes in these samples was also compared with those that are meat-based because these types of samples contained high amounts of vegetables in addition to the meat. The results showed that poultry meat-based samples exhibited higher counts than those reached by vegetable-based samples. However, no significant differences were found when compared with beef-based samples (*p* = 0.303). In fact, there were remarkably high counts of bacteria harboring *tet* genes in vegetable-based samples, although the presence of antimicrobial resistant bacteria in vegetable products has also been detected by other authors [24,35,36]. The high rates of Tc-resistant bacteria in this type of sample could be explained by the presence of Tc residues and *tet* genes transmitted to vegetable crops through different routes, such as manure, sewage and surface or irrigation waters [37,38], which may have human health consequences.

The amount of Tc residue equivalents was analyzed with a receptor assay to verify whether there was any correlation between the amount of residues and the amount of bacteria harboring the *tet*(A) and/or the *tet*(B) genes in the baby foods (Table 2). Almost all of the samples had less than 50 µg kg^−1^ Tc residue equivalents and the analyzed genes were present in almost all of them. Although there was no correlation between the amount of residues and the amount of resistant bacteria, it was observed that the samples with detectable amounts of Tc residue equivalents were also positive for the presence of *tet* genes. In contrast, when the presence of the genes was not detected, the sample was also negative for the presence of Tc residues. In addition, both types of farming methods, conventional and organic, were compared, and 37% of organic poultry meat-based samples were positive *versus* 21% of conventional samples. In the case of organic beef-based samples, 27% were positive in contrast with 18% from conventional production. Surprisingly, the higher average of residues was obtained in samples of vegetables and in organic baby foods, especially in beef-based samples, in which a total of 5 samples were over the maximum residue limit allowed for Tc in muscle in the European Community [39]. It is a well-known fact that the bibliography gives importance to the spread of resistances to tetracycline due to the use of antimicrobials in treatment of the animals. However, the use of tetracyclines in agriculture for the treatment of some fruits [40] could play an important role to explain the results shown by us for TetA and TetB genes in vegetable-based baby foods. It would be of interest to investigate the use of a “waiting time”, or other cleaning strategy, before using the vegetable products to avoid the presence of residues or resistant bacteria in food.

## 4. Conclusions

Regarding the results obtained for the *tet*(A) and *tet*(B) genes, it can be concluded that this study may serve to assess the quality of raw baby food material before being sterilized and to help the producers to know if organic baby foods are actually better than conventional products. Both *tet* genes were present in all types of organic baby foods. Therefore, we cannot conclude that organic formulas are better than conventional formulas for the studied parameters. Furthermore, the amount of *tet* genes also suggests that they are widely distributed, especially *tet*(A), in foods of animal origin as well as in vegetable-based products.
